# Detection of Various Traditional Chinese Medicinal Metabolites as Angiotensin-Converting Enzyme Inhibitors: Molecular Docking, Activity Testing, and Surface Plasmon Resonance Approaches

**DOI:** 10.3390/molecules28207131

**Published:** 2023-10-17

**Authors:** Qixin Wu, Yue Jiao, Mingzhu Luo, Jingyi Wang, Jingzhe Li, Yanyan Ma, Changzhen Liu

**Affiliations:** Experimental Research Center, China Academy of Chinese Medical Sciences, Beijing 100700, China

**Keywords:** angiotensin-converting enzyme 1, surface plasmon resonance, the metabolite of traditional Chinese medicine, affinity

## Abstract

Angiotensin-converting enzyme 1 (ACE1) is a peptide involved in fluid and blood pressure management. It regulates blood pressure by converting angiotensin I to angiotensin II, which has vasoconstrictive effects. Previous studies have shown that certain compounds of natural origin can inhibit the activity of angiotensin-converting enzymes and exert blood pressure-regulating effects. Surface Plasmon Resonance (SPR) biosensor technology is the industry standard method for observing biomolecule interactions. In our study, we used molecular simulation methods to investigate the docking energies of various herbal metabolites with ACE1 proteins, tested the real-time binding affinities between various herbal metabolites and sACE1 by SPR, and analyzed the relationship between real-time binding affinity and docking energy. In addition, to further explore the connection between inhibitor activity and real-time binding affinity, several herbal metabolites′ in vitro inhibitory activities were tested using an ACE1 activity test kit. The molecular docking simulation technique’s results and the real-time affinity tested by the SPR technique were found to be negatively correlated, and the virtual docking technique still has some drawbacks as a tool for forecasting proteins′ affinities to the metabolites of Chinese herbal metabolites. There may be a positive correlation between the enzyme inhibitory activity and the real-time affinity detected by the SPR technique, and the results from the SPR technique may provide convincing evidence to prove the interaction between herbal metabolites and ACE1 target proteins.

## 1. Introduction

Hypertension is a cardiovascular syndrome characterized by persistent systolic blood pressure of more than 130 mmHg or diastolic blood pressure of at least 80 mmHg. The primary clinical symptom is increased systemic circulation artery pressure [1]. The long-term development of chronic hypertension, one of the most prevalent chronic diseases in humans, can seriously harm human health by causing structural and functional changes to several target organs, including the heart, kidneys, brain, and eyes [2,3,4]. Studies have shown that in addition to coronary heart disease and stroke, hypertension is an important risk factor for heart failure, atrial fibrillation, chronic kidney disease, aortic syndrome, and dementia, and it is known as an “invisible killer” affecting human health [5,6,7].

The pathogenesis of hypertension is complex. Activation of the renin-angiotensin-aldosterone system (RAAS), sodium and water retention in the kidneys, aberrant ion transport across the cell membrane, and insulin resistance are currently thought to be contributing factors [8,9,10,11]. Among them, the RAAS is a complex network regulatory system consisting of various enzymes and short peptides, with the key product angiotensin II as the core [12]. Angiotensin II can cause vasoconstriction, raising blood pressure by binding to angiotensin receptors [13], increasing peripheral sympathetic nerve tension by promoting norepinephrine release from sympathetic nerve endings, and increasing sympathetic nerve discharge activity [14]. This peptide is converted from angiotensin I by the ACE1 that exists both in the membrane-binding form and in the secreted form. It is widely distributed in the lungs, brain, heart, liver, kidneys, intestine, and other target organs and is presented in a variety of other cell types, such as absorptive epithelial cells, neuroepithelial cells, and male germ cells, as well as the vascular endothelial cells of the placenta [15,16]. Nowadays, angiotensin-converting enzyme inhibitors (ACEI) have become one of the most common treatments for preventing and controlling hypertension. Captopril, Enalapril, Fosinopril, and other ACEI are vigorously utilized in clinical practice. However, accompanying therapeutic benefits of these medications, several serious side effects, i.e., cough, vascular edema, renal insufficiency, and hyperkalemia are also exhibited [17,18,19,20]. They are prompting researchers and clinicians to seek novel, more effective and safer ACEI agents.

Natural compounds have the characteristics of diverse structures, single components, wide sources, clear targets, and low toxicity. In recent years, numerous studies on ACEI derived from plants have been conducted, in which many natural compounds were found having the potent ACEI activity [21]. Several plant-derived extracts, including Coumarins, Anthocyanins Delphinidin, Cyanidin-3-O-samubiosides, Quercetin, and Flavonoids, have reportedly been shown to inhibit the activity of the angiotensin-converting enzyme [22,23,24,25,26,27,28,29,30,31,32,33,34]. Therefore, natural inhibitors for the angiotensin-converting enzyme might be a very promising source of more effective and safer ACEI candidates. Several techniques screening natural inhibitors have been created in the past years, including magnetic bead immobilization, enzyme immobilization, colorimetric, spectrophotometric, fluorescence, high-performance liquid chromatography, etc. [35,36,37]. In some of these methods, the reagents are complicated, and the catalyst-induced reactions are easily affected by temperature. In some other methods, the laborious and boring experimental process may easily lead to unrepeatable or false results. In addition, the methods above cannot provide the equilibrium dissociation constant of the interaction between inhibitors and target proteins, which is important for the subsequent structure–activity relationship research and the structural transformation of compounds.

As one of the most advanced unlabeled biosensing technologies, SPR technology has become an authority technique in the field of compound–protein interaction detection. It is suited to sensitively detect the interaction between two molecules in real time. The basic principle of SPR is that in the case of total internal reflection, the incident light causes the resonance of the thin metal plasma, which results in the emission of light at a certain angle with an energy that is nearly zero [38]. The weight of the chip surface changes after the analyte binds to it, changing the refractive index, and this results in a reflection that is sensitive to the binding of molecules on the sensor chip [39,40]. Owing to its numerous advantages such as stable, sensitive, real-time, and label-free detection of intermolecular conjugation and dissociation processes, SPR technology is widely utilized in pathogen detection [41], biomarker detection [42,43,44], screening of active compounds [45,46], antibody screening [47], and other research. In recent years, the advantages of SPR for the detection of ligands between target proteins and small molecules have been fully exploited [48,49]. The quick and efficient detection of small molecules with high-affinity ligands, though, is gradually becoming difficult. The vast majority of conventional lead compound discovery techniques demand time-consuming, extensive effectiveness verification. Our team used SPR to evaluate the binding affinity of inhibitors to target proteins, combining the advantages of this technique, which include accuracy, efficiency, and the need for minimal assay material, for the effective evaluation of compounds and highly automated large-scale assays during the pre-drug screening stage [50,51]. Furthermore, the SPR instrument’s varied properties allowed us to investigate the affinity of the same target proteins for different types of TCM metabolites.

At first, computer simulation technology was utilized to estimate the drug ACE1 binding modes and their affinities, evaluate the potential biological activity of natural compounds, and compare the outcomes of analytical screening. The technology of SPR was used to detect the real-time affinity of inhibitors with target proteins. Finally, the inhibitory activities of selected compounds were then determined in vitro using the angiotensin-converting enzyme activity assay. This study’s multi-angle drug screening method will supplement previous ACEI evaluation methods, especially in the way of compensating for the inadequacies of existing inhibitor screening methods in examining binding kinetics. It may contribute to ACEI screening, design, and modification in the future, as well as support the development of natural ACEI.

## 2. Results

### 2.1. Molecular Docking Analysis

**Figure 1:**
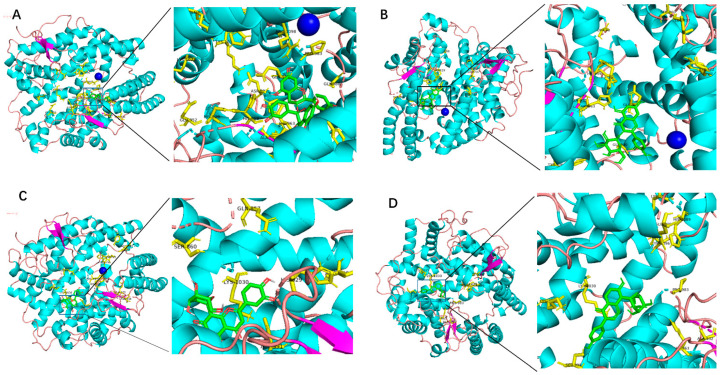
Molecular docking models between different TCM metabolites and the C-terminal of ACE1. Yellow represents the peptide segment that interacts with TCM metabolites, dark blue spheres represent zinc ions, green represents different TCM metabolites, and light blue dotted lines represent the interacting chemical bond. (**A**) Procyanidin, (**B**) Isoquercitrin, (**C**) Orientin, (**D**) Puerarin.

**Figure 2:**
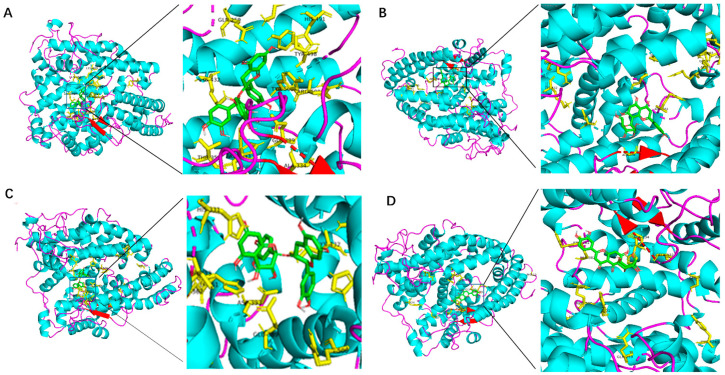
Molecular docking models between different TCM metabolites and the N-terminal of ACE1. Yellow represents the peptide segment that interacts with TCM metabolites, green represents different TCM metabolites, and light purple dotted lines represent the interacting chemical bond. (**A**) Procyanidin, (**B**) Orientin, (**C**) Isoquercitrin, (**D**) Homeorienin.

Through computer simulation of molecular docking, we analyzed the docking energy between traditional Chinese medicine (TCM) metabolites and ACE1, evaluated the potential biological activity of candidate compounds, and achieved the goal of low-cost screening in a short period. The binding energy between the C-terminal or the N-terminal of the ACE1 protein and the candidate compounds and the amino acids involved in the interaction are shown in Table 1 and Table 2. Among the 10 TCM metabolites, Procyanidin, Isoquercitrin, Orientin, and Homeorienin have high binding energy values at the C and N ends of the ACE1, and the docking energy is lower than −8 kcal/mol, whereas Resveratrol and two terminal ends of the ACE1 have relatively low binding energy values, and the docking energy is high at −7.5 kcal/mol. The docking models between some candidate compounds and the C-terminal molecules of ACE1 are shown in Figure 1. Procyanidin, Isoquercitrin, Orientin, and Puerarin, respectively, bind to zinc-binding catalytic sites and can interact with zinc ions through van der Waals forces. The docking model between Procyanidin, Orientin, Isoquercitrin, and Homeorienin and the N-terminal molecules of ACE1 is shown in Figure 2. The docking simulations of other TCM metabolites are shown in the Appendix A.

### 2.2. Preparation of the Recombinant sACE1 Protein

**Figure 3:**
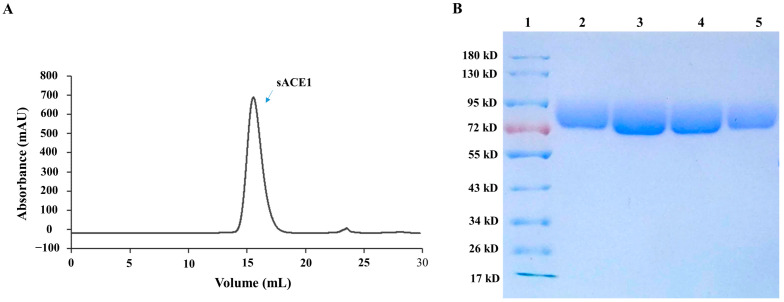
Purification products and SDS-PAGE analysis of the sACE1 protein. (**A**) Purification of ACE1 recombinant protein by volume exclusion chromatography. (**B**) Affinity chromatography purification of ACE1 by SDS-polyacrylamide gel electrophoresis: (1) standard protein, (2) protein collected by molecular sieve at 15.0–15.5 mL, (3) protein collected by molecular sieve at 15.5–16.0 mL, (4) protein collected by molecular sieve at 16.0–16.5 mL, (5) protein collected by molecular sieve at 16.5–17.0 mL.

To further investigate the interactions between TCM metabolites and the target ACE1 protein, we prepared the recombinant soluble C domain of ACE1 (sACE1), which is more important for blood pressure regulation than the N domain and completely accounts for the blood pressure regulation activity of ACE [52,53], by the molecular cloning and recombinant protein expression and purification method. sACE1 cDNA was inserted into eukaryotic expression vector pcDNA3.1, and the recombinant protein was expressed by HEK293 cells, which were transiently transfected with the constructed plasmid. After purification by Ni-NTA affinity chromatography, the purified recombinant sACE1 was obtained after size-exclusion chromatography (Figure 3A). The result of SDS-PAGE showed that the molecular weight of the obtained protein was about 80 k_D_, which was consistent with the theoretical size, and the purity of the obtained protein should be higher than 90% (Figure 3B).

### 2.3. SPR Determination between TCM Metabolites and sACE1

**Figure 4:**
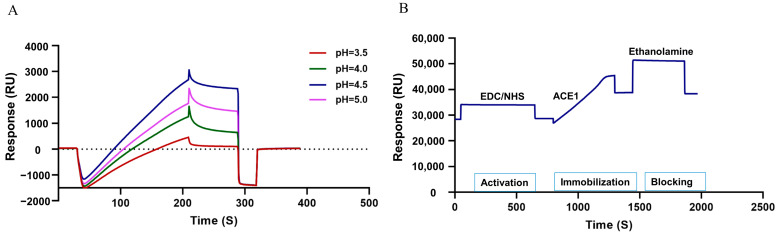
The ACE1 sensor chip surface operating condition. (**A**) Response of immobilization assay at different pH levels. (**B**) Amino coupling of ACE1 with the wizard model.

We then analyzed the interaction between TCM metabolites and sACE1 by SPR technology. Firstly, the chips were prepared. Recombinant sACE1 was diluted with four different pH values (pH concentrations of 3.5, 4.0, 4.5, 5.0) of sodium acetate to achieve the optimal coupling condition. Under the condition of pH 4.5, the maximum amount of protein was enriched on the chip (Figure 4A). Then, we activated the carboxyl groups on the chip surface with the mixture of NHS (0.2 mmol/L) and EDC (0.5 mmol/L) and injected the sACE1 protein, which was diluted with sodium acetate (pH 4.5) to covalently immobilize this protein on the chip. The unoccupied activated carboxyl groups on the chip were further blocked by ethanolamine. After the complete coupling process, the immobilized ACE1 protein level of channel 2 was about 10,000 Ru (Figure 4B).

**Figure 5:**
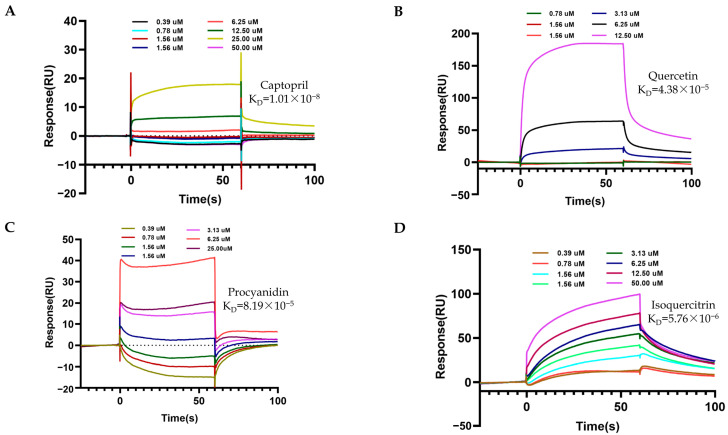
The affinity between TCM metabolites or Captopril and ACE1. A series of concentrations (0.39–50.0 µM) of metabolites was tested to obtain the affinity between ACE1 and metabolites by kinetic analysis. (**A**) The fitted curve for different concentrations of Captopril binding to ACE1. (**B**) The fitted curves for different concentrations of Quercetin binding to ACE1. (**C**) The fitted curves for different concentrations of Procyanidin binding to ACE1. (**D**) The fitted curves for different concentrations of Isoquercitrin binding to ACE1.

**Figure 6:**
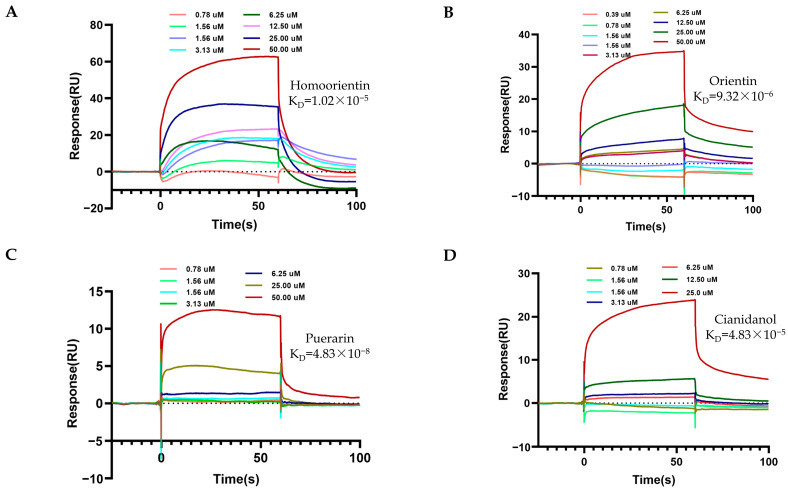
The affinity between TCM metabolites and ACE1. A series of concentrations (0.39–50.0 µM) of metabolites was tested to obtain the affinity between ACE1 and metabolites by kinetic analysis. (**A**) The fitted curve for different concentrations of Homoorientin binding to ACE1. (**B**) The fitted curves for different concentrations of Orientin binding to ACE1. (**C**) The fitted curves for different concentrations of Puerarin binding to ACE1. (**D**) The fitted curves for different concentrations of Cianidanol binding to ACE1.

The candidate TCM metabolites diluted to a series of concentrations were then injected onto the surface of the coupled ACE1 protein chip at a flow rate of 30 uL/min for 180 s to achieve full binding between the TCM metabolites and the protein. Next, a dissociation stage for 300 s, in which the running buffer flowed through the chip at 30 uL/min to allow the bound TCM metabolites to detach themselves from the protein, was arranged to evaluate the parameters of dissociation. As shown in Table 3, Figure 5 and Figure 6, the compounds such as Quercetin, Procyanidin, Isoquercitrin, Homoorientin, Orientin, Puerarin, and Cianidanol were all bound to sACE1 on the chip in a dose-dependent manner, similar to the positive reagent captopril. The value of the equilibrium dissociation constant (K_D_) between compounds and ACE1 was calculated, in which Isoquercitrin had the strongest affinity for sACE1 (K_D_ 5.76 × 10^−6^ M), and the positive reagent captopril and Cianidanol had the weakest affinity (K_D_ 4.83 × 10^−5^ M).

### 2.4. ACE1 Inhibitory Activity of Various Candidate TCM Metabolites

**Figure 7:**
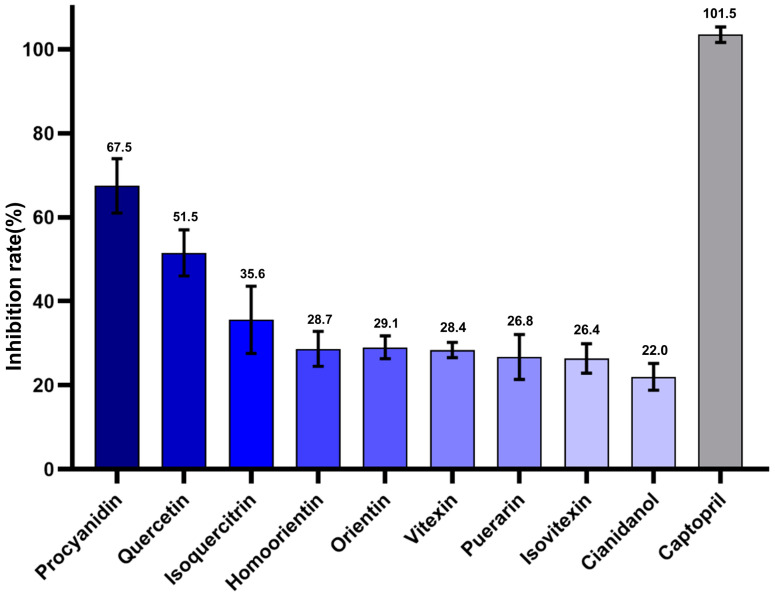
Effects of different TCM metabolites on angiotensin-converting enzyme activity. The inhibitory activities (%) of captopril or different TCM metabolites were evaluated at 1 uM or 25 uM concentrations, respectively. The inhibition rate of captopril was taken as 100%, and the relative inhibition rate of other TCM metabolites was calculated. The positive control group was captopril, and the negative control was an equal concentration of DMSO.

The in vitro inhibitory activities of each of the aforementioned potential compounds were assessed using a chemical kit. Procyanidin, Quercetin, and Isoquercitrin demonstrated significant inhibitory actions, with inhibitory activities of 67.5%, 51.5%, and 35.6%, respectively. Other compounds, including Homoorientin, Orientin, Vitexin, Puerarin, Isovitexin, and Cianidanol, all demonstrated more than 20% inhibitory efficacy (Figure 7).

### 2.5. Correlation Analysis

**Figure 8:**
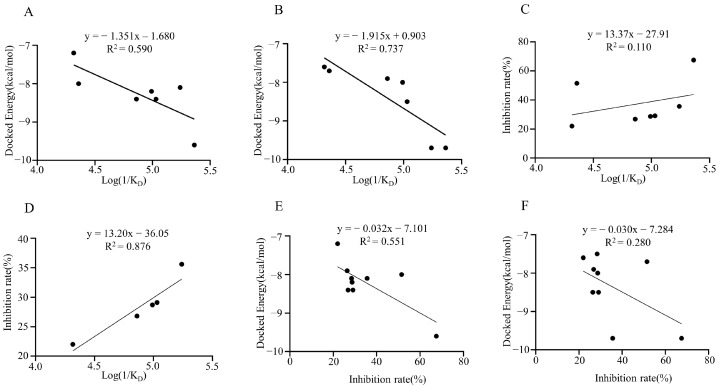
The correlation analysis between molecular docking, SPR detection, and enzyme inhibitory activity. (**A**) The correlation between log (1/K_D_50) and docked energy of C-terminal ends of ACE1. (**B**) The correlation between log (1/K_D_50) and docked energy of N-terminal ends of ACE1. (**C**) The correlation between log (1/K_D_50) and inhibition rate (%). (**D**) The correlation between log (1/K_D_50) and inhibition rate (%) after removing Procyanidins and Quercetin data. (**E**) The correlation between inhibition rate (%) and docked energy of C-terminal ends of ACE1. (**F**) The correlation between inhibition rate (%) and docked energy of N-terminal ends of ACE1.

We then correlated the results from the molecular docking with those from the SPR analysis, respectively. The binding energy deduced from the molecular docking was used as one of the relational values, and the K_D_ value measured from the SPR analysis was used as another parameter. The relationship between the ACE-C-terminal and TCM metabolites was evaluated as molecular docking energy = −1.351 log (1/K_D_) −1.680 (R^2^ = 0.59, *p* < 0.05) (Figure 8A). The relationship between the ACE-N-terminal and TCM metabolites was evaluated as molecular docking energy = −1.915 log (1/K_D_) + 0.903 (R^2^ = 0.7367, *p* < 0.05) (Figure 8B). 

We then executed a correlation analysis between the SPR-detected affinity and the TCM metabolites′ enzyme inhibitory activity. The relationship between the enzyme inhibition rate and the experimental value log (1/K_D_) was analyzed using the enzyme inhibition rate of a TCM metabolite as one of the relationship values, and the K_D_ value obtained from the experiment as another parameter. The relationship was evaluated as enzyme inhibition rate = 13.37 log (1/K_D_) −27.91 (R^2^ = 0.1101, *p* = 0.4671) (Figure 8C). Interestingly, after removing Procyanidins and Quercetin data, which had a high degree of dispersion, the relationship between them was enzyme inhibition rate = 13.20 log (1/K_D_) −36.05 (R^2^ = 0.8759, *p* < 0.05) (Figure 8D). Additionally, the association between the ACE-C-terminal and TCM metabolites was calculated as molecular docking energy = −0.03162 enzyme inhibition rate −7.1 (R^2^ = 0.5507, *p* < 0.05) (Figure 8E). The relationship between ACE-N terminus and TCM metabolites evaluated as molecular docking energy = −0.03019 enzyme inhibition rate −7.28 (R^2^ = 0.2799, *p* = 0.1431) (Figure 8F). 

## 3. Discussion

Inhibitors of the angiotensin-converting enzyme have become the first-line medications for the clinical prevention and treatment of hypertension. With centuries of study and application, an increasing number of individuals are opting to treat hypertension with herbal remedies, given their fewer negative effects. In recent years, researchers have increased their focus on the naturally occurring ACEIs in the plant kingdom, and a number of techniques have emerged for the detection of ACEI activity, such as LC-ES-MS (Liquid Chromatography-Electrospray Ionization Mass Spectrometry), UPLC-MS (Ultra-Performance Liquid Chromatography-Electrospray Ionization Mass Spectrometry), and UV-Spectrophotometry. However, most of the previous assays rely on laborious techniques like bioactivity and substrate assessments, and the exact affinity characteristics of TCM metabolites have not been documented [29,54]. In order to better understand the medical functions of the TCM metabolites and their interactions with the target protein ACE1, in this study, we summarized previously reported TCM metabolites that may have ACE1 inhibitory activities and analyzed the docking energies between ACE1 and the metabolites, determining their binding affinity. In addition, we measured the enzyme inhibitory activities of the metabolites, which may provide new evidence for the following investigations of the anti-hygroscopic properties.

According to previous research, although the two structural domains of the extracellular region of ACE1, the N-terminal domain and the C-terminal domain, share a similar structure, the latter domain is more crucial for direct blood pressure regulation [40,41]. In this study, we prepared the soluble C-terminal domain of tACE1 (sACE1) by recombinant DNA technology to determine the binding of TCM metabolites to the protein, investigating the potential mechanism of metabolites involved in controlling blood pressure. In our experiment, pH 4.5 was identified as the optimal coupling pH value for cross-linking the recombinant protein on the chip, and the well-immobilized protein provided a good foundation for further SPR analysis.

In our experiment, we screened TCM metabolites with docking energies less than 10^−7^ with both the C-terminal and N-terminal domain of ACE1 by molecular docking simulations, in which Resveratrol was screened out as a qualified metabolite. However, in subsequent enzyme activity tests, we discovered that Resveratrol did not inhibit ACE1 in vitro, and it had no direct affinity activity with ACE1 by SPR assays, suggesting that although resveratrol can lower blood pressure in rat models and in some patients [55], it may not act through direct inhibition of angiotensinase activity, which is consistent with previous reports by Olszanecki R [25]. The correlation analysis showed that despite the reasonableness of the relationship between molecular docking-simulated proteins and TCM ligands, it was still inadequate as a tool for predicting protein–TCM metabolite affinity.

Isovitexin, like bradykinin B2 receptor antagonists, has been proven in studies to reduce blood pressure via vasodilating blood arteries [56]. Our experimental results, combined with this report, suggested that the antihypertensive effect of Vitexin may not be driven by directly binding to ACE1 target proteins, and its functional mechanism still remains to be explored.

Our study showed that Procyanidin and Quercetin performed better than most of the other TCM metabolites both in terms of molecular docking simulation results and enzyme inhibitory activity species. According to the analysis of previous studies, Procyanidin is a polyphenolic compound with a polyhydroxy structure, which leads to enhanced interactions with target proteins and reduced molecular docking energies. Meanwhile, ACE1 is a protein located in the membrane that has the characteristic of being easily enriched for macromolecules [24,30,31]. Based on this, polyphenolic compounds like procyanidin may result in greatly enhanced interactions between metabolites and the enzyme. Similarly, the polyhydroxy group, a structural characteristic of quercetin, specifically the oxo-functional group at the 4-position and hydroxyl groups at positions 3 and 4 of the B-ring, could result in the greatly enhanced interaction and the significantly increased enzyme inhibitory activity [57]. Thus, it is not surprising that Procyanidin and Quercetin have outstanding enzyme inhibitory activity.

Fluorescence-based biosensors frequently employ labeling techniques that demand harsh detection conditions and produce unstable results [58]. Lakowicz and his team created surface plasmon-enhanced emission, fluorescent probes on metal films, to overcome the inefficiency and experimental false positives of conventional fluorescence detection techniques. This greatly boosted the sensitivity and selectivity of the detection system. SPR biosensor technology, which similarly utilizes surface plasma, an electromagnetic wave, is now the gold standard for detecting biomolecular interactions [59,60,61]. Due to the sharp increase in its detection accuracy and sensitivity, SPR has recently attracted more attention for its use in small-molecule drug research, including various procedures like lead compound discovery, pharmacokinetic studies, ADME (adsorption, distribution, metabolism, and excretion) prediction, and target identification [62]. In our experiment, the unique advantages of SPR technology for label-free detection of the interaction between TCM metabolites and sACE1 proteins were exploited. On the one hand, the real-time quantitative binding affinity assay uses relatively less material and has the potential to be developed into a high-throughput assay in the future. On the other hand, this method is not limited by the toxicity of the drug, which may be a serious trouble for the traditional cellular and animal activity assays and may provide a new approach for the activity assay of lead compounds.

The overall study combines the low-cost identification of potentially active compounds by virtual screening, the enzyme bioactivity tests verifying the direct inhibitory activity of TCM metabolites, and the SPR binding assay focusing on the kinetic parameters of the binding between ACE proteins and herbal ligands. To the best of our knowledge, this is the first study to determine the affinity between ACEI-type herbal metabolites and ACE1 proteins, providing evidence for the exploration of relevant pharmacological mechanisms of action of each metabolite. Additionally, it serves as a starting point for the optimization of herbal substances, which will contribute to the development of trustworthy and efficient ACEIs in later research.

## 4. Materials and Methods

### 4.1. Instruments and Materials

These included the following: Biacore T200 system (GE Healthcare, Uppsala, Sweden), transfection kit (KAIRUI biotech, Zhuhai, China), AKTA avant 25 protein purification system (GE Healthcare, Uppsala, Sweden), Superdex200 10/300GL volume exclusion chromatography column (GE Healthcare, Uppsala, Sweden), electrophoresis equipment (Bio-Rad, Hercules, CA, USA), microplate reader (PerkinElmer, Waltham, MA, USA), ACE1 activity test kit (Sigma, Shanghai, China), metabolite of TCM (Ruifensi, Chengdu, China), The Amino Coupling Kit, 10 × HBS-EP buffer solution, CM5 Sensor Chip, and Ni-NTA affinity beads were purchased from GE Healthcare (Braunschweig, Germany). Zinc chloride and other chemical reagents (Sigma, Shanghai, China).

### 4.2. Molecular Docking Simulation

To preliminarily determine the ACE1 inhibitory activity of selected TCM metabolites, we used the AutoDock 1.5.6 program for molecular docking simulation. The secondary structure of TCM metabolites was obtained from the PubChem database (PubChem (nih.gov accessed on 28 September 2022) and was optimized by Chem office Ultra 14.0 software (Cambridge Soft Co., Boston, MA, USA) to minimize energy. The three-dimensional structures of the C-terminal domain (7q4c) and N-terminal domain (7q49) of human ACE1 were obtained from the PDB database (http://www.rcsb.org/pdb/home/home.do accessed on 28 September 2022). The solvent and small-molecule ligand structure of human ACE1 were removed by Pymol, and AutoDock 1.5.6 software was used to set the radius and coordinate position of the active pocket of the protein. TCM metabolites with a docking energy less than 10^−7^ were screened, and molhttpecular docking was performed using Pymol-validated docking simulation to demonstrate the specific amino acids involved in the interaction between the target protein and different TCM metabolites [63].

### 4.3. Construction of the Expression Plasmids

The plasmid containing human full-length ACE1 cDNA (HG11598) was purchased from Sino Biological Inc. (Beijing, China). The cDNA coding for the soluble C domain of ACE1 (Ile640-Ala1231) was obtained by PCR amplification from full-length ACE1 cDNA using the designed primers 5′-ATCTGCGGCCGCTATAGACCTGGTGACTGATGAGGCTG-3′ and 5′-TAGACTCGAGTTAAGCGGAGTTCGGCGTCCAGTTG-3′ and was inserted into the cloning sites of restriction enzymes Not I and Xho I of the modified expression vector pcDNA3.1, which was previously inserted in a cDNA sequence coding the HSA signaling peptide and the his-tag peptide (GCCACCATGAAGTGGGTAACCTTTATTTCCCTTCTTTTTCTCTTTAGCTCGGCTTATTCC GGATCCCACCATCACCACCATCATCACCACCATCAC) between the cloning sites of restriction enzymes Kpn I and Not I. The expression product is the recombinant sACE1 with an N-terminal his-tag. The constructed plasmid was confirmed by DNA sequencing.

### 4.4. Expression and Purification of sACE1 Recombinant Protein

The constructed plasmid was transiently transfected into suspended HEK293 cells according to the protocol of the transfection kit. Briefly, suspended HEK293 cells were grown in KOP293 medium in an Orbital Shaker at 110 RPM at 37 ℃ and 5% CO_2_, and approximately 100 mL of cells at the exponential stage (about 2~4 × 10^6^ cells/mL) were used to be transfected. About 100 ug of sterile plasmid DNA was added into 5 mL KPM (transfection buffer) in a sterile centrifuge tube, and 500 μL of TA-293 transfection reagents were added to 5 mL KPM in another sterile centrifuge tube. After mixing, the diluted transfection reagent was added to the diluted plasmid and mixed thoroughly. After incubation for 10 min at room temperature, the mixture was added to cells, and the transfected cells were incubated in an orbital shaker at 110 RPM at 37 ℃ and 5% CO_2_. After six days, the supernatant of the cultured medium was collected by centrifugation (4 ℃, 10,000× *g*, 10 min) and filtered through a 0.45 uM filter membrane. The sACE1 recombinant protein contained in the supernatant was purified by Ni-NTA affinity chromatography. Briefly, the supernatant was diluted with twice the volume of phosphate buffer solution (PBS, 135 mmol/L NaCl, 2.7 mmol/L KCl, 1.5 mmol/L KH_2_PO_4_, 8 mmol/L K_2_HPO_4_, pH7.4), and imidazole was added to a final concentration of 20 mmol/L. The diluted cell supernatant flowed slowly through the Ni-NTA affinity column three times to ensure the protein was fully bound to affinity beads. The unbound component in the supernatant was washed away by washing buffer 1 (50 mM Tris-HCl, 50 mM imidazole, 0.5 M NaCl, pH 8.0) and washing buffer 2 (50 mM Tris-HCl, 100 mM imidazole, 500 mM NaCl, pH 8.0), and then the bound protein was eluted by elution buffer (50 mM Tris-HCl, 500 mM imidazole, 500 mM NaCl, pH 8.0). The eluted product was further purified by size-exclusion chromatography buffered with PBS to obtain the purified sACE1 recombinant protein. The purity and molecular weight of the prepared protein were examined by SDS-PAGE electrophoresis assay.

### 4.5. SPR Sensor Measurements

The SPR measurements were carried out using a Biacore T200. Prepared sACE1 was immobilized onto the surface of the CM5 chip to be used as the stationary phase. Briefly, protein was diluted to 20 μg/mL with immobilization buffer and then covalently coupled on channel 2 of the CM5 chip using an amine coupling kit according to the manufacturer’s protocol. Channel 1 without cross-linked protein was used as a blank control. Gradient concentrations (50 uM, 25 uM, 12.5 uM, 6.25 uM, 3.125 uM, 1.5625 uM, 0.78 uM, 0.39 uM) of the TCM metabolites were diluted in the running buffer (PBS, with 5% (*v*/*v*) DMSO, 3 mM EDTA, and 0.05% (*v*/*v*) surfactant P20, pH = 7.4) and injected into both channels at a flow rate of 30 μL/min as mobile phases. The contact time and dissociation time were set to 60 s and 300 s, respectively, and then the extra wash with 50% DMSO was applied. An analytical buffer without TCM metabolite was used to correct the effect brought by the solvent. All steps were performed at 25 °C, and data were retrieved and analyzed by Biacore T200 Evaluation Software, assuming the steady-state affinity analysis model.

### 4.6. ACE Inhibition Measurement

Referring to the approach of Guru Prasad Sharma et al. [64], TCM metabolite compounds with lower docking energy in the molecular docking simulation were further tested for ACE inhibition activity in vitro using an ACE1 activity test kit. Different TCM metabolites were diluted with the detection buffer to a concentration of 25 uM. A total of 10 uL of the dilutions was added into a black 96-well plate containing 80 uL of substrate and 10 uL of standard substance. The fluorescence produced by the reaction mixtures was measured by microplate reader with an excitation wavelength of 320 nm and an emission wavelength of 405 nm. The activity inhibition rates of different TCM metabolites on the substrate were calculated using captopril as a positive control and the reaction buffer as a negative control. Three replicates of all tested samples were used.

### 4.7. Analysis of Inhibition Rate Data

Using the values of fluorescence intensity change of the buffer as the negative control and the values of fluorescence intensity change of captopril as the positive control, the activity inhibition rates of TCM metabolites with different concentrations were calculated using the following equation:

Inhibitory activity (%) = [1− ((A_a_ − A_0_) − (A_b_ − A_0_))/((A_c_ − A_0_) − (A_d_ − A_0_))] × 100% (1)

A_0_ is the fluorescence intensity containing buffer alone. A_a_ is the fluorescence intensity of the mixture containing TCM metabolite dilution and substrate at the 45th min. A_b_ is the fluorescence intensity of the mixture containing TCM metabolite dilution and substrate at the 0th min. A_c_ is the fluorescence intensity of the mixture containing substrate at the 45th min, and A_d_ is the fluorescence intensity of the mixture containing substrate.

## 5. Conclusions

In this work, we initially screened for the more active ACE1 inhibitors using research from the literature and molecular docking virtual screening. We employed the SPR technique to assess the real-time affinity of various TCM metabolites and tested the enzymatic activity. Among the 12 TCM metabolites that inhibited angiotensinase I in vitro by more than 20% and all had less than 10–7 molecular docking energy with ACE-1 protein, 9 TCM metabolites were demonstrated to have better affinities with the ACE1 target protein in vitro. In addition, analysis of the results of molecular docking simulations and real-time affinity revealed a significant correlation between them, and the molecular docking simulations could be used as a prediction tool for protein–herbal medicine metabolite affinity to save experimental costs. Finally, we would like to show that the combined screening of ACEI activity by multiple modalities may provide a better solution for the pharmacodynamic screening of more toxic lead compounds.

## Figures and Tables

**Table 1:** Molecular interactions between ACE inhibitors and the C-terminal of ACE1.

ACE Inhibitors	Docked Energy (kcal/mol)	Specific Amino Acids for Hydrophobic Interactions
Procyanidin	−9.6	GLN-857,HIS-1089,TYR-1096,ARG-1098,GLU-987,PRO-983,GLY-980,HIS-963,ASN-642,TYR-936,ASP-934,ALA-932,HIS-963,ASP-991,GLU-952,HIS-959
Quercetin	−8.0	ASN-787,SER-795,ALA-932,ARG-1098,ASP-697,GLY-980,LYS-1025,GLU-960,ALA-937,CYS-946,LYS-1030
Isoquercitrin	−8.1	GLU-986,GLY-980,TYR-936,HIS-963,ASP-934,ALA-932,HIS-959,HIS-929,LEU-737,SER-998,SER-860
Resveratrol	−7.3	HIS-1089,SER-795,HIS-963,ASP-934
Homoorientin	−8.2	HIS-959,ASP-991,LYS-1025,LYS-1030,SER-998,GLU-987,HIS-963,ASP-934,LYS-1087,ASP-1029
Orientin	−8.4	SER-860,GLN-857,LYS-1030,ASP-991,ARG-1098,TRP-796,SER-795,HIS-929,GLY-980,GLU-979,ASP-934,ARG-700
Isovitexin	−7.9	GLU-979,HIS-929,HIS-959,SER-931,ALA-932,LYS-1030
Puerarin	−8.4	LYS-1025,SER-998,LYS-1030,HIS-963,ALA-932,PRO-983,GLU-738,TRY-1096,HIS-1089,LYS-1087
Vitexin	−8.1	GLN-857,HIS-1081,HIS-1030,HIS-957,ASP-991,GLY-980
Cianidanol	−7.2	LYS-1030,SER-998,HIS-959,HIS-929,SER-931,SER-1093,SER-795,ALA-932,GLU-699,TRY-936,TYR-970

**Table 2:** Molecular interactions between ACE inhibitors and the N-terminal of ACE1.

ACE Inhibitors	Docked Energy (kcal/mol)	Specific Amino Acids for Hydrophobic Interactions
Procyanidin	−9.7	GLN-259,LYS-489,HIS-491,TYR-498,LYS-432,THR-358,TYR-186,ASP-393,HIS-361,GLU-389,ALA-334,ARG-500,TYR-501,TYR-498
Quercetin	−7.7	TYR-501,TYR-498,HIS-491,GLN-259,GLY-382,ARG-381,ARG-90,TRY-369, ALA-334,HIS-361,SER-357
Isoquercitrin	−9.7	GLN-259,LYS-432,HIS-491,ARG-500,GLU-389,ASP-393,HIS-331,HIS-361,SER-333,GLU-362,TYR-369,ARG-381,ARG-500,HIS-491,TRP-198,THR-97,TYR-186,TYR-111
Resveratrol	−7.0	ARG-381,GLN-259,LYS-489,ARG-500,GLN-81,GLN-444
Homoorientin	−8.0	HIS-365,ALA-334,ALA-332,SER-357,GLY-382,ARG-381,ARG-500, TYR-501,TYR-424,HIS-491,GLN-259
Orientin	−8.5	ARG-381,PRO-385,ALA-334,ASP-393,TRP-201,ASN-203,SER-100,THR-97, TRP-201,GLN-259,LYS-432,ARG-96,GLY-93,ARG-89
Isovitexin	−8.5	ALA-334,TYR-369,PRO-385,ARG-500,TYR-501,SER-35,GLN-62,TYR-24,ARG-90,ALA-101,ARG-96,TYR-197,SER-200,ASN-203,GLN-81
Puerarin	−7.9	HIS-365,ALA-334,HIS-331,TYR-424,ARG-90,THR-97,ALN-81,GLN-259,SER-200
Vitexin	−7.5	GLN-259,TYR-498,TRP-201,TYR-501,HIS-331,TYR-186,TYR-338,ARG-108
Cianidanol	−7.6	SER-260,GLU-431,TYR-498,HIS-491,SER-200,TYR-338,SER-61,ARG-96

**Table 3:** The affinity between metabolites and ACE1.

Compounds	K_D_ (M)
Captopril	1.01 × 10^−8^
Procyanidin	4.34 × 10^−6^
Quercetin	4.38 × 10^−5^
Isoquercitrin	5.76 × 10^−6^
Homoorientin	1.02 × 10^−5^
Orientin	9.32 × 10^−6^
Puerarin	1.38 × 10^−5^
Cianidanol	4.83 × 10^−5^

**Figure A1:**
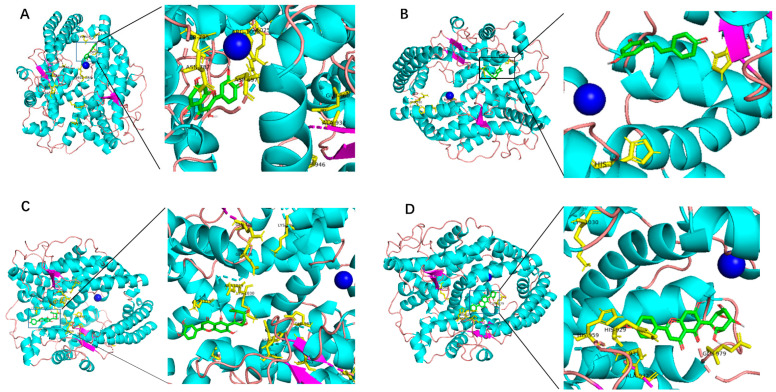
Molecular docking models between different TCM metabolites and the C-terminal of ACE1. Yellow represents the peptide segment that interacts with TCM metabolites, dark blue spheres represent zinc ions, green represents different TCM metabolites, and light blue dotted lines represent the interacting chemical bond. (**A**) Quercetin, (**B**) Resveratrol, (**C**) Homoorientin, (**D**) Isovitexin.

**Figure A2:**
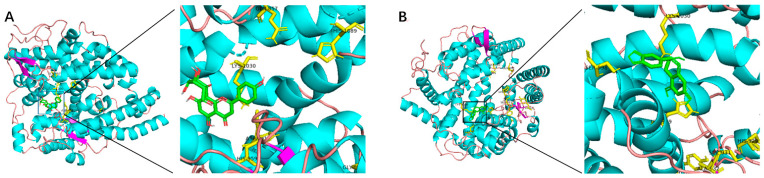
Molecular docking models between different TCM metabolites and the C-terminal of ACE1. Yellow represents the peptide segment that interacts with TCM metabolites, dark blue spheres represent zinc ions, green represents different TCM metabolites, and light blue dotted lines represent the interacting chemical bond. (**A**) Vitexin, (**B**) Cianidanol.

**Figure A3:**
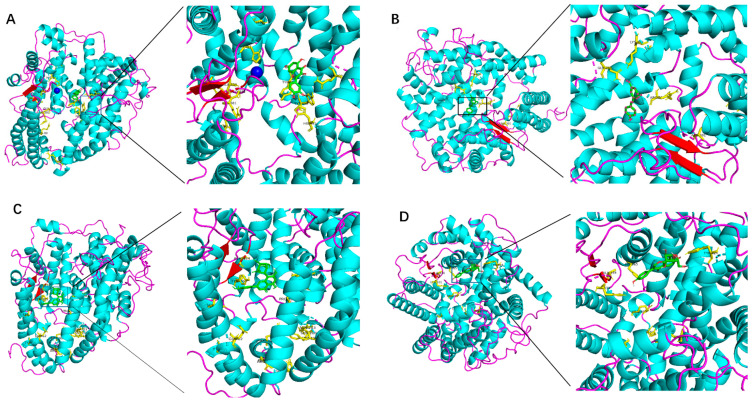
Molecular docking models between different TCM metabolites and the N-terminal of ACE1. Yellow represents the peptide segment that interacts with TCM metabolites, green represents different TCM metabolites, and light purple dotted lines represent the interacting chemical bond. (**A**) Quercetin, (**B**) Resveratrol, (**C**) Isovitexin, (**D**) Puerarin.

**Figure A4:**
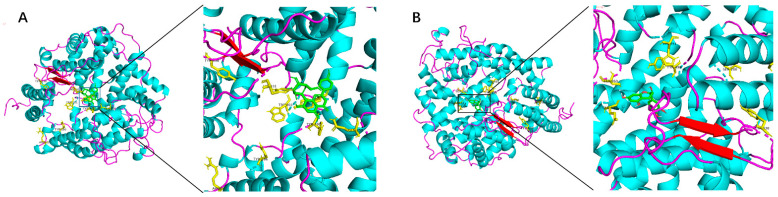
Molecular docking models between different TCM metabolites and the N-terminal of ACE1. Yellow represents the peptide segment that interacts with TCM metabolites, green represents different TCM metabolites, and light purple dotted lines represent the interacting chemical bond. (**A**) Vitexin, (**B**) Cianidanol.
